# The Potential Role of Tumor Microorganisms in Remodeling Tumor Biomechanical Signals and Application Strategies in Tumor Therapy

**DOI:** 10.3390/microorganisms14040807

**Published:** 2026-04-01

**Authors:** Xiang Li, Jing Chang, Qingxin Xia, Jinxiao Yan, He Zhang, Hui Yang

**Affiliations:** 1 School of Life Sciences and Technology, Northwestern Polytechnical University, Xi’an 710129, China; 2 Engineering Research Center of Chinese Ministry of Education for Biological Diagnosis, Treatment and Protection Technology and Equipment, Northwestern Polytechnical University, Xi’an 710129, China; 3 Research Center of Special Environmental Biomechanics & Medical Engineering, Northwestern Polytechnical University, Xi’an 710129, China; 4 Department of Clinical Nutrition, Affiliated Cancer Hospital of Zhengzhou University & Henan Cancer Hospital, Zhengzhou 450008, China; 5 Department of Pathology, Affiliated Cancer Hospital of Zhengzhou University & Henan Cancer Hospital, Zhengzhou 450008, China

**Keywords:** tumor microorganism, TME, biomechanics, therapy

## Abstract

The tumor microenvironment (TME) is a highly dynamic and heterogeneous system composed of tumor cells, stromal cells and non-cellular components that collectively govern tumor initiation, progression, and metastasis. Beyond host-derived components, accumulating evidence has established that microorganisms are integral constituents of the TME. These tumor-associated microbes not only affect tumor cells directly but also reshape the TME. Importantly, microbial-driven remodeling of the TME is accompanied by changes in its biomechanical properties. These alterations introduce a biophysical dimension of the TME that operates alongside biochemical signaling. Studies have shown that certain microorganisms reshape mechanotransduction pathways within tumor cells. Such biomechanical alterations enhance tumor cell adaptability to shear stress, promote survival in circulation, and facilitate invasion and colonization at distant sites. Although direct evidence linking microbes to specific biomechanical changes in tumors remains limited, these preliminary insights point to a largely unexplored yet highly promising frontier. This review explores the physical properties of the TME and delineates the association between microorganisms and the dynamics of these physical signals. It then examines strategies for leveraging microorganisms in tumor therapy. Understanding these interactions may reveal novel therapeutic targets to harness microbial influences and inhibit tumor progression.

## 1. Introduction

Oncology is undergoing a transformative shift in our understanding of tumor etiology and therapy. Traditionally, cancer research has focused on genetic and cellular anomalies as primary drivers of tumorigenesis [1,2]. However, recent advances have highlighted the critical role of microorganisms within tumors, ushering a new era in tumor microbiome research [3]. Beyond well-established oncogenic viruses [4], a diverse array of bacteria and fungi has been identified within the tumor microenvironment (TME) [3,5]. These microbes are not passive bystanders but active participants that modulate the tumor ecosystem [6]. Notably, specific microbial profiles have been associated with particular cancer types [7], suggesting potential involvement in tumorigenesis [8,9]. Moreover, tumor-resident microbes influence tumor progression, immune evasion, and therapeutic responses [10].

These microorganisms affect tumor biology through mechanisms such as altering the metabolic landscape and modulating the immune microenvironment [11]. They also present opportunities as diagnostic biomarkers and therapeutic targets, offering new directions for cancer management [12]. Integrating host genetics with microbial profiling may pave the way for personalized oncology [13]. However, despite its promise, this field remains nascent, with unanswered questions: What mechanisms underlie microbial contributions to tumor progression? How can we harness the tumor microbiome to improve therapeutic efficacy?

As tumors grow rapidly, they disrupt their native environment and establish a unique TME, characterized by distinct physical signal changes within the tumor [14]. Recent technological advancements have revolutionized our ability to measure and analyze the physical properties of tumor cell and the TME [15], shedding light on how cancer cells sense and respond to physical microenvironment. Increasingly, we recognize the mechanical roles of both cells and the extracellular matrix (ECM) in tumor proliferation and migration [16]. Additionally, some studies have also explored microbial involvement in tumor development. For instance, Fu et al. identified intracellular bacteria within cancer tissues that contribute to metastasis and colonization [17]. Although the link between these microorganisms and the tumor’s physical microenvironment remains underexplored, this finding offers a new dimension to tumor progress.

This review summarizes the biomechanical properties of the TME. Subsequently, we review the impact of tumor-resident microorganisms on both the TME and its biomechanical properties. Finally, we outline emerging strategies for leveraging the tumor microbiota in cancer therapy. In sum, this review aims to provide novel insights into how tumor microbiota regulate the physical microenvironment, thereby facilitating the development of innovative strategies for basic research and clinical intervention.

## 2. Tumor Microenvironment: A Biomechanical Perspective

**Figure 1:**
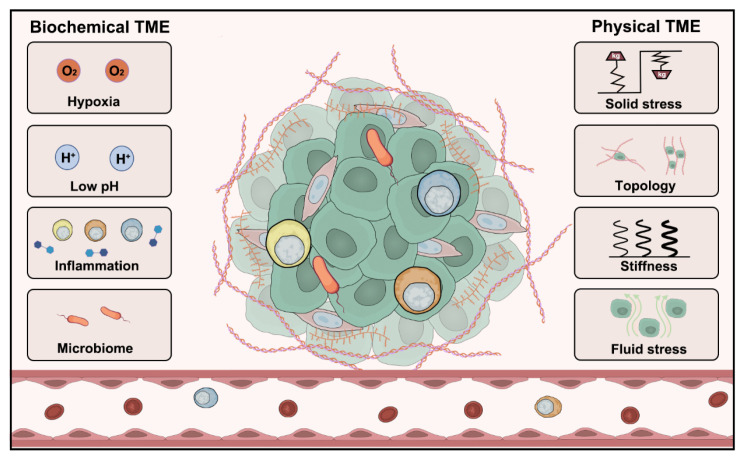
The changes in biochemical and physical properties of TME. Biochemical TME: Characterized by hypoxia, acidic pH conditions, chronic inflammation, and microbiome interactions, creating a metabolically hostile niche that drives tumor progression and therapy resistance. Physical TME: Defined by mechanical forces including solid stress (compressive/tensile), ECM topology, tissue stiffness, and fluid shear stress, which collectively regulate tumor cell invasion, metastasis, and drug delivery efficiency.

The dynamic interaction between tumor cells and the TME is a central regulator of tumor progression and metastasis. These interactions encompass not only significant biochemical changes such as hypoxia, acidosis, cytokine signaling, and microbial composition, but also significant alterations in physical properties (Figure 1) [18]. Driven by rapid proliferation, tumor growth often outstrips the blood supply, leading to oxygen insufficiency (hypoxia). Hypoxia triggers adaptive mechanisms including angiogenesis and metabolic reprogramming [19]. Meanwhile, TME acidosis driven by lactic acid accumulation from anerobic metabolism, not only affects tumor cell survival and proliferation but also enhances invasiveness and metastatic potential [20]. The crosstalk among various cell types and molecular signals within the TME contributes to the complexity of its biochemical characteristics. In terms of physical properties, the TME differs significantly from healthy tissues. Changes such as increased matrix stiffness can influence tumor cell morphology, function and behavior.

These dynamic biochemical and physical alterations are deeply interrelated. Biochemical signals can remodel the ECM, thereby altering tissue stiffness, while physical changes can, in turn, influence biochemical stability such as the activation of latent TGF-β1 by matrix stiffening in hepatocellular carcinoma [21]. Therefore, a comprehensive understanding of both the biochemical and physical properties of the TME and their interplay is essential for elucidating tumor development and metastasis, and for identifying new therapeutic strategies.

### 2.1. Biochemical Properties of TME

The biochemical microenvironment of tissue includes hypoxia, acidosis, cytokines, unique microbial community, and other factors (Figure 1). The TME is characterized by unique features such as hypoxia and low pH [22], largely due to abnormal vasculature and enhanced glycolysis [23,24]. These conditions activate adaptive responses in cancer cells, promoting aggressive phenotypes and contributing to therapeutic resistance. However, it is important to note that certain stressors within the TME can also induce cell death in at least a subset of tumor cells [25], highlighting the complex and some-times paradoxical roles of these microenvironmental cues in tumor biology. Moreover, the immunosuppressive microenvironment, which is dominated by regulatory T cells and myeloid-derived suppressor cells, further complicates the TME landscape [26]. A thorough understanding of these biochemical complexities is essential for designing targeted therapies that disrupt pro-tumorigenic signaling and enhance treatment efficacy.

### 2.2. Biomechanical Properties of TME

In parallel with its biochemical features, the physical microenvironment of tumor also plays a critical role in cancer progression. As shown in Figure 1, ECM provides structural support and regulates cellular behavior through its composition and stiffness [27]. Solid stress, from proliferating tumor cells leads to tissue compression, impairing vascular function and drug delivery [28]. Fluid stress, driven by elevated interstitial fluid pressure (IFP), further alters the physical microenvironment [29]. Additionally, the three-dimensional architecture of the TME shapes cellular interactions and functional responses [30].

Studies of the physical properties of tumors have revealed a complex interplay between tumor cells and the TME. Tumor cells exhibit distinct mechanical traits that contribute to their malignancy, while the physical characteristics of the TME significantly influence cancer progression. These properties must be considered at multiple scales. At the single-cell level, tumor cells are generally softer than healthy cells [31], whereas at the tissue level, solid tumors are typically stiffer than healthy tissues.

#### 2.2.1. Cellular Biomechanical Features

In the TME, tumor cells function as both recipients and generators of physical signals. As the main drivers of cancer progression, they exhibit distinct physical characteristics that significantly influence their behavior and contribute to malignancy. Recent advances have illuminated the pivotal role of these physical traits in cancer biology. These physical cues include stiffness, deformability, and contractility.

Stiffness: A hallmark physical property of tumor cells is their altered stiffness relative to normal cells. Typically, they exhibit reduced stiffness, enhancing their ability to infiltrate adjacent tissues and circulate through the bloodstream [32]. Cross et al. reported that metastatic cancer cells isolated from pleural effusions of lung, breast, and pancreatic cancer patients were over 70% softer than benign cells from the cavity [33]. One major contributor to this reduced stiffness is the dynamic remodeling of the cytoskeleton. In tumor cells, cytoskeletal changes, including actin filament reorganization, can alter cell stiffness. Alicia et al. examined the cytoskeletal architecture of breast cancer and normal breast cell lines using peak–force modulation atomic force microscopy and immunofluorescence confocal microscopy [34]. They found that actin stress fibers localized in the apical regions of healthy cells, but restricted to basal regions in tumor cells. These basal actin fibers did not enhance tumor cell stiffness.

Deformability: Another key physical trait of tumor cells is their increased deformability, which enables them to traverse confined tissue spaces and invade surrounding tissues. Even with matrix metalloproteinases (MMPs), enlarging effective ECM pore size [35], cells must deform considerably to pass through gaps roughly ten times smaller than their own diameter [36]. Notably, more deformable breast cancer cells demonstrate enhanced invasiveness in vitro [16], and in situ analyses of human breast biopsies show that invasive cancer cells are more deformable than those from benign lesions [37]. Chen et al. further advanced this understanding using a zebrafish model, demonstrating that undifferentiated tumor cells can squeeze between endothelial cells and penetrate ECM pores via highly deformable mechanisms [38]. This finding may offer a potential target for therapeutic strategies aimed at slowing or preventing extravasation and metastasis. The enhanced deformability of tumor cells has far-reaching implications for cancer biology. For instance, tumor cells with impaired display even greater deformability than controls [39], facilitating invasion, vascular entry, and distant dissemination. This mechanical phenotype is tightly linked to the invasive potential of tumor cells.

Contractility: Tumor cells also exhibit elevated contractility driven by the activation of myosin motors. Myosin II binds to actin filaments to generate contractile forces, with its activity regulated by myosin light chain kinase (MLCK) and Rho-associated protein kinase (ROCK) [40]. This increased contractility enables tumor cells to exert mechanical forces that facilitate migration and tissue remodeling. Laklai et al. demonstrated that enhanced contractility in tumor cells correlates with increased invasiveness and metastatic potential [41]. Their study highlighted that the loss of transforming growth factor-beta (TGF-β) signaling activates a G protein-coupled receptor-mediated JAK/STAT3 pathway, which in turn stimulates ROCK1. This leads to elevated cellular contractility, ECM remodeling, and local tissue stiffening, thereby promoting focal adhesion maturation and facilitating tumor progression and invasion.

Moreover, in vitro three-dimensional studies suggested that, beyond protease-mediated degradation, tumor cells also utilize a force-dependent invasion mechanism to traverse the basement membrane [42]. These studies showed that cells coordinate global volume expansion with localized myosin-driven contraction to apply shear forces on the basement membrane, generating openings that permit cell migration [43]. The findings highlighted how contractile forces drive collective invasion through the basement membrane, which is an essential step in metastatic dissemination.

#### 2.2.2. Physical Cues in TME

The TME is a complex milieu in which tumor cells interact with stromal cells, the ECM, and diverse physical factors. These physical cues include solid stress, tissue stiffness, fluid stress, and topology.

Solid stress: Solid stress in the TME, comprising compressive and tensile forces, arises from factors such as increased matrix deposition, tumor expansion, and shear stress [44]. Proliferation and infiltration of tumor cells, along with ECM accumulation, contribute to tumor growth, which in turn compresses and displaces surrounding structures, generating solid stress [45,46]. Certain ECM components, such as hyaluronic acid, can absorb water and swell, further increasing solid stress [47]. Solid stress significantly influences cancer cell behavior. As stress intensifies, it can compress or collapse blood and lymphatic vessels [48,49,50], inducing hypoxia within the TME [49,51], which compromises the efficacy of chemotherapy, radiotherapy, and immunotherapy [22,52,53]. In addition to these indirect effects, solid stress may directly enhance tumor cell migration and invasion—as observed in breast cancer and contribute to neuronal loss and neurological dysfunction in the brain [46,49,54].

Stiffness: Stiffness is an intrinsic material property of the TME. Increased tissue stiffness is one of the most readily detectable features of tumors and has long been used in tumor diagnostics, particularly in manual assessments such as breast palpation and mammography [55]. Across various cancer types—including breast cancer [56], pancreatic cancer [57], liver cancer [58], and prostate cancer [59], malignant tumors typically exhibit higher stiffness than benign tumors. This characteristic is influenced by various factors, including the ECM composition, and cell–matrix interactions.

Changes in ECM components within tumor tissues, such as enhanced collagen deposition and elevated fibronectin expression, contribute significantly to increased stiffness [60,61]. Additionally, TGF-β is commonly upregulated in the ECM [62]. Cancer-associated fibroblasts (CAFs), owing to their abundance, robust secretory capacity, and ability to assemble ECM components. Elevated TGF-β1 levels promote CAF activation, further stimulating ECM deposition and promoting tumor progression [63].

Cell–matrix interactions are critical regulators of stiffness within the TME. These interactions, mediated by adhesion molecules such as integrins regulate how tumor cells sense and respond to matrix cues. When microenvironmental stiffness changes, integrins detect mechanical signals, undergo conformational changes, and relay these signals to the cytoskeleton via proteins like talin [64]. Integrin activation triggers downstream signaling pathways, including focal adhesion kinase and the Hippo pathway transcription factors Yes-associated protein (YAP) and TAZ. Activated YAP further enhances CAF activity [65,66,67].

Fluid stress: Fluid stress differs fundamentally from solid stress and has a distinct origin. It comprises microvascular fluid stress, IFP, and shear stress [29]. Tumor blood vessel leakage and deformation increase geometric resistance to blood flow, resulting in poor perfusion and inadequate delivery of nutrients and oxygen [68]. Concurrently, these abnormalities elevate IFP in the tumor core to levels significantly higher than in normal tissue [69]. The elevated IFP drives fluid from the tumor center toward the periphery, generating shear stress. This shear stress can activate fibroblasts [70], modulate endothelial cell behavior [71], transport pro-angiogenic factors to promote further blood and lymphatic vessel formation [70,72], induce MMPs activation and cellular motility [73], and initiate processes that facilitate tumor cell migration and invasion [74,75]. IFP is associated with treatment response and prognosis in several cancers, including cervical cancer, lymphoma, melanoma, and lung cancer [76]. Shear stress has been reported to promote liver cancer stem cell migration by activating focal adhesion kinase (FAK) and extracellular signal-regulated kinase 1/2 (ERK1/2) pathways [77]. Additionally, autophagy induction via the integrin/cytoskeleton pathway enhances HepG2 cell migration and invasion [78], and shear stress in lymphatic vessels has been shown to activate YAP1, thereby promoting cancer cell migration [79].

Topology: Topology refers to cell geometry and matrix architecture. The TME consists of cellular and ECM components. In normal tissues, these components are arranged in a highly organized manner. However, during tumor progression, excessive cell proliferation disrupts this order by altering solid stress, leading to microstructural changes in the TME. These changes are particularly evident in the altered geometry of tumor cells. Simultaneously, the composition and structure of ECM components are also modified.

Tumor cells exhibit deformability and contractility, both of which contribute to altered cell geometry. Beyond these mechanical changes, water efflux can reduce cell volume, influencing tumor cell stiffness and stem cell fate [80]. The nucleus geometry is also affected by overall cell shape, which in turn regulates gene expression [81,82]. Deformation of cells and nuclei can reorganize perinuclear actin and microtubule networks, alter chromatin arrangement, and drive YAP/TAZ nuclear translocation [83].

CAFs regulate the deposition of collagen and other ECM components in the TME, thereby altering its stiffness. At the same time, CAFs influence the cross-linking of matrix components through the secretion of enzymes such as lysyl oxidase and MMPs [84,85]. Beyond cross-linking, the alignment of ECM fibers, particularly collagen, serves as physical tracks that direct cancer cell migration. Tumor cells tend to migrate along aligned collagen fibers [86], whereas reticular collagen in the breast impedes invasion. In contrast, densely packed collagen fibers, oriented perpendicular to the tumor boundary, promotes invasion [87].

The topology of blood vessels in the matrix also undergoes substantial remodeling. Overexpression of vascular endothelial growth factor (VEGF) and other pro-angiogenic factors induces neovascularization, leading to structural abnormalities at both macro- and micro levels [88,89,90]. As tumorigenesis progresses, tumor microvessels become dilated, tortuous, and cystic, exhibiting irregular patterns of connectivity and branching [91,92]. At the cellular level, endothelial cells (ECs) in tumor vasculature display disorganized and irregular morphologies. While mature ECs are stabilized by adherens junctions, involving vascular endothelial (VE)-cadherin [93], tumor-associated ECs often show disrupted or overlapping junctions, reduced VE-cadherin expression, and elongated cytoplasmic protrusions [94,95].

## 3. The Emerging Role of Intratumoral Microbiota

### 3.1. Spatial Organization of Tumor Microorganisms

The human body harbors diverse microbial communities collectively known as the human microbiome, which plays an essential role in maintaining health and immune homeostasis [96]. However, microbiome composition varies considerably due to factors such as genetic background, environment, diet, and lifestyle [97], resulting in significant inter-individual microbiome differences. Thus, this variability is also reflected in TME.

Different types of cancer harbor distinct tumor-associated microbial communities [3]. Nejman et al. conducted a comprehensive characterization and analysis of the microbiota in the TME, studying 1526 samples from seven tumor types and their adjacent normal tissues. This study not only confirmed that different tumors possess distinct microbial compositions but also found that breast cancer exhibits the highest microbial abundance and diversity. Similarly, Jiang et al. observed that bacterial abundance in tumor tissues of non-smoking lung adenocarcinoma patients was significantly higher than in adjacent non-tumor tissues [98].

The microbial composition differs between tumor tissues and non-tumor tissues. At the end of the last century, the discovery of *Helicobacter pylori* overturned the long-held view of the stomach as a sterile organ. In addition to *Helicobacter pylori*, several other genera *Enterococcus*, *Lactobacillus*, *Carnobacterium*, *Glutaminibacter*, and *Fusobacterium* were found to be increased in gastric tumor tissues compared to those from non-gastric tumor patients [99]. Another set of experiments showed that the gastric microbiota of gastric tumor patients is enriched with bacterial genera and species commonly residing in the oral cavity, including symbiotic or opportunistic bacteria such as *Neisseria*, *Alloprevotella*, *Aggregatibacter*, *Porphyromonas endodontalis*, and *Streptococcus mitis* [100]. In addition to organs directly exposed to the external environment, microbial composition also differs between tumor tissues and adjacent non-tumor tissues in relatively closed organs such as the breast. Compared to adjacent non-tumor tissues, breast cancer tissues show significant enrichment of four bacterial phyla: *Proteobacteria*, *Firmicutes*, *Bacteroidetes*, and *Actinobacteria* [101,102].

The bacteria and their associated functions within tumors are influenced by tumor type and subtype, patient lifestyle, and response to immunotherapy. Hieken et al. collected sterile normal breast tissues from women with benign or malignant disease and analyzed the matrix composition [103]. Their results showed that alpha and beta diversity of the tissue microbiome varied with fat and fibrosis content. Differences in the abundance of *Firmicutes*, *Bacilli*, *Bacillales*, *Staphylococceae* and the genus *Staphylococcus* were associated with fat levels. A similar pattern was observed for fibrosis, which was linked to variations in *Firmicutes*, *Spirochaetes*, *Bacilli*, *Bacillales*, *Spirochaetales*, *Proteobacteria RF32*, *Sphingomonadales*, *Staphylococcaceae*, and the genera *Clostridium*, *Staphylococcus*, *Spirochaetes*, *Adlercreutzia Actinobacteria*.

The microbial composition also varies across different stages of cancer progression. There are differences in microbial diversity and richness compared to superficial gastritis, atrophic gastritis, and intestinal metaplasia, suggesting microbial dysbiosis during gastric cancer [104]. Notably, the operational taxonomic units (OTUs) corresponding to *Parvimonas micra*, *Dialester pneumonites*, *Slackia exigua*, *Peptostreptococcus stomatis*, *Prevotella intermedia*, *Fusobacterium nucleatum*, *Prevotella oris*, and *Catonella mori* were significantly enriched in gastric cancer compared to precancerous stages.

### 3.2. TME Reprogramming Induced by Microorganisms

Advances in analytical and sequencing technologies have enabled more precise characterization of the TME microbiome, thereby facilitating deeper exploration of its functional impact on the host. Microorganisms impact the TME, through multiple mechanisms: stimulating angiogenesis to remodel tumor vasculature, modulating the immune landscape to alter immune cell infiltration and function and directly interacting with tumor cells to influence their behavior and metastatic potential.

#### 3.2.1. Microbial Influence on Immune Response

Research has shown that bacterial peptides can mimic tumor antigens or directly activate immune cells. While cancer cells often evade immune detection by concealing their antigens, exogenous bacterial peptides exhibit greater immunogenic potential than tumor-derived antigens [105]. In melanoma metastases, bacterial peptides presented by human leukocyte antigen (HLA) molecules on tumor cells and infiltrating immune cells demonstrate their utility as tumor-specific antigens. B cells displaying bacterial peptide-HLA complexes can activate tumor-infiltrating T cells, promoting IFN-γ secretion and highlighting their immunogenicity [106]. Microbes may also introduce antigens that cross-react with tumor-specific antigens a phenomenon termed “molecular mimicry”. In melanoma patients, higher homology between tumor neoantigen and microbial epitopes correlates with better clinical outcomes [107]. Aurélie Fluckiger’s team identified cross-reactive T cells in cancer patients that recognize both tumor and microbial antigens. They discovered a phage-derived antigen, which shares epitopes with the tumor-associated antigen PSMB4. In mouse models, MHC-I- presented measured protein elicited specific CD8 T cells that targeted PSMB4-expressing tumor cells, enhancing PD-1 blockade efficacy and prolonging survival [108].

Additionally, microbes can directly induce tumor cell death, releasing antigens that stimulate immune responses. Pathogen-specific targeting within the TME triggers tumor cell lysis, releasing tumor antigens, damage-associated molecular patterns (DAMPs), and pathogen-associated molecular patterns (PAMPs). This recruits peripheral immune cells to the TME or reactivates pre-existing anti-tumor immunity [109,110]. Furthermore, microbes themselves can act as potent immune adjuvants, fostering an inflammatory environment that bolsters anti-tumor immunity.

Microbial-derived metabolites can be recognized by tumor cells and immune cells, modulating the tumor immune microenvironment. Receptors for these metabolites are expressed on both cancer cells and tumor-infiltrating immune cells. Short-chain fatty acids (SCFAs)—such as acetate, propionate, and butyrate—are produced by gut anaerobic bacteria during dietary fiber fermentation. In normal intestinal epithelial cells, SCFAs suppress pro-tumor inflammation. For instance, chronic excessive intestinal inflammation in the early stage promotes colonic epithelial hyperproliferation, DNA damage, and malignant transformation, thereby fueling tumor development. However, butyrate elevates interleukin-10 (IL-10) and retinoic acid levels in the intestinal microenvironment, promoting regulatory T cells (Treg) differentiation and proliferation, thereby inhibiting pro-tumor inflammation [111].

Microbes also regulate the tumor immune microenvironment by targeting immune checkpoints, including PD-1, CTLA-4, TIM-3, LAG-3, T cell immunoreceptor with Ig and ITIM domains (TIGIT), and carcinoembryonic antigen-related cell adhesion molecule 1 (CEACAM1). *Fusobacterium nucleatum* suppresses natural killer (NK) cell and cytotoxic T cell activity through Fap2 interactions with TIGIT or CEACAM1 [112,113]. Similarly, *Helicobacter pylori* uses its outer membrane protein HopQ to bind CEACAM1 inhibiting immune cells [114]. HopQ-CEACAM1 interaction facilitates the virulence factor CagA translocation and IL-8 release, driving gastric epithelial damage, inflammation, and tumorigenesis [115].

Notably, the crosstalk between intratumoral microbiota and the TME is reciprocal rather than unidirectional. Intratumoral microbes actively shape the tumor immune microenvironment. They modulate immune cell infiltration, inflammatory signaling, and immune checkpoint expression. Meanwhile, the immunosuppressive TME in turn creates a permissive niche for microbial colonization. Antitumor immunity is weakened in tumors. Cytotoxic lymphocytes become exhausted, and immunosuppressive cell populations increase. These changes together reduce the host antimicrobial defense and microbial clearance, which support the stable residence and proliferation of intratumoral bacteria. For instance, *Listeria monocytogenes* exploits the myeloid-derived suppressor cells within the TME as a carrier [116]. The bacterium infects these immunosuppressive cells. In this way, the bacterium not only gains safe passage into the tumor core but also effectively evades elimination by the host immune system, achieving stable colonization. This bidirectional interaction forms a self-reinforcing cycle. It contributes to sustained microbial colonization, tumor-promoting inflammation, and progressive cancer development.

#### 3.2.2. Microbial Influence on Tumor Angiogenesis

Furthermore, microbes secrete proangiogenic factors that promote blood vessels formation, supplying tumors with nutrients to growth and progression. In colorectal cancer (CRC), *Clostridium butyricum* downregulates methyltransferase-like 3 expression, reducing vimentin and vascular endothelial growth factor receptor 2 (VEGFR2) levels [117]. It inhibits epithelial–mesenchymal transition (EMT) and vasculogenic mimicry, processes that provide tumors with nutrients and a microenvironment conducive to proliferation and invasion.

Another key aspect of microbial regulation in tumor angiogenesis involves modulating VEGF expression in host cells, thereby influencing tumor vascularization. For instance, YYC-3, a metabolite secreted by *Lactobacillus plantarum*, can suppress CRC cell metastasis by inhibiting the VEGF-MMP2/9 signaling pathway [118]. Since VEGF and MMP2/9 are critical drivers of tumor blood vessel formation, this microbial intervention highlights their role in shaping the tumor vascularization.

#### 3.2.3. Microbial Interactions with CAFs and ECM

Beyond tumor cells, the second-most frequently infected cell population within primary tumors resides in collagen-rich regions (15%), likely representing CAFs [119]. As central regulators of collagen-rich tumor ECM dynamics, CAFs facilitate collective tumor migration and are detectable among circulating tumor cells. Emerging evidence reveals that specific microbes can activate CAFs within the TME. This activation of CAFs is a significant event during cancer progression. Microbial components, such as bacterial lipopolysaccharides (LPS), act as potent CAF activators by binding Toll-like receptors (TLRs) on their surface. TLR engagement triggers intracellular signaling cascades that upregulate proinflammatory cytokines (e.g., IL-6) [120], growth factors (e.g., TGF-β), and ECM-remodeling g enzymes (e.g., MMPs). In liver cancer, LPS also induces hepatic progenitor cell differentiation into myofibroblasts, fostering an inflammatory microenvironment [121]. These myofibroblasts derived from hepatic progenitor cells further amplify tumorigenesis by secreting IL-6 and TNF-α, which activate Ras signaling while suppressing p53 in hepatic progenitor cells. Consequently, this dual perturbation promotes aberrant proliferation and malignant transformation, ultimately driving tumor initiation.

## 4. Modulation of Tumor Biomechanics by Microorganisms

Beyond their established roles in remodeling the TME, tumor-associated microbiota have recently attracted attention for their influence on the physical microenvironment, particularly its biomechanical properties. Such microbiota can directly modulate the mechanical characteristics of tumor cells and other cellular components, while also indirectly reshaping tissue-scale mechanics through reorganization of the extracellular matrix, tissue architecture, and force transmission. These biomechanical alterations may further drive malignant progression and therapy resistance, offering a novel biomechanical perspective on microbiota–host interactions.

### 4.1. Reshaping Cellular Mechanophenotype

**Figure 2:**
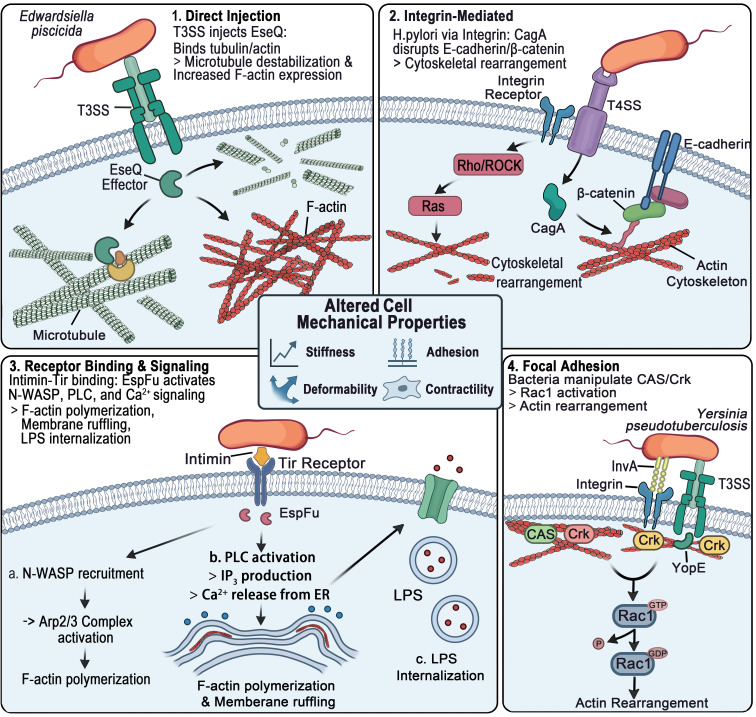
The potential ways of microorganisms in reshaping cellular mechanophenotype. (1) T3SS injects effector proteins into host cells, activating associated signaling pathways; (2) *H. pylori* disrupt E-cadherin-β-catenin-actin linkages through virulence factors (CagA); (3) Bacterial secreted Intimin binds to the host receptor Tir, triggering multiple downstream signaling pathways; (4) Bacteria manipulate host focal adhesion proteins, CAS and Crk, to activate Rac1, initiating actin cytoskeletal rearrangements.

Although existing studies have demonstrated that microorganisms can reorganize the cytoskeleton and modulate cellular responses to vascular shear stress, there remains a lack of sufficient research that directly links defined cellular mechanophenotypes to microbial influences. Most studies have focused on microbe-induced cytoskeletal remodeling and the activation of associated signaling pathways. Nevertheless, previous work has also suggested multiple potential ways through which microorganisms may directly or indirectly influence cellular mechanophenotypes (Figure 2). Direct contact between microorganisms and host cells can impact cell stiffness. Microorganisms may indirectly regulate cytoskeletal dynamics and mechanical behavior by secreting cytokines or other soluble factors.

**Figure 3:**
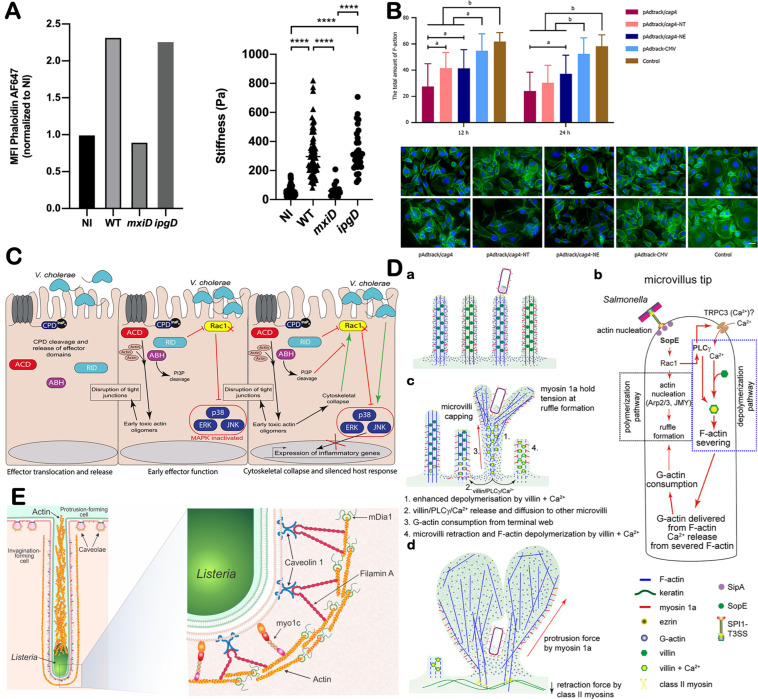
The studies and proposed models of microorganisms in reshaping tumor cellular mechanophenotype. (**A**) Increased stiffness associated with increased filamentous actin in *Shigella*-invaded cells. Adapted with permission from Ref. [122]. Copyright 2020, Wiley-VCH. **** *p* < 0.0001. (**B**) Quantification of the F-actin cytoskeleton with FITC phalloidin stain. Adapted with permission from Ref. [125]. Copyright 2024, Baishideng. ^a^ *p* < 0.05, ^b^ *p* < 0.01, Scale bar = 25 μm. (**C**) Model of interplay between the multiple functions of the MARTXVc toxin. Adapted with permission from Ref. [126]. Copyright 2020, American Association for the Advancement of Science. (**D**) Model for a proposed sequence of manipulations of host cell actin by *Salmonella*. (**a**) After STM adheres to MV, the SPI1-T3SS translocates effector proteins into host cells. (**b**) SopE activates Rac1, which promotes F actin polymerization and ruffle formation via WASP/WASH–Arp2/3, and also activates PLCγ villin to sever MV F actin and release Ca^2+^. (**c**) Ca^2+^ and PLCγ-villin complexes diffuse to adjacent MV to trigger further F-actin depolymerization, while ruffle formation consumes cytoplasmic G-actin and accelerates MV disassembly. (**d**) Extensive F-actin depolymerization and G-actin depletion lead to MV collapse. Myosin 1a supports ruffle extension, resulting in brush border loss and fully formed ruffles. Adapted with permission from Ref. [127]. Copyright 2023, Frontiers Media. (**E**) Proposed model of mDia1 and caveola-associated F-actin-binding proteins at *L. monocytogenes* membrane invaginations. Adapted with permission from Ref. [128]. Copyright 2021, American Society for Microbiology.

Direct contact between microorganisms and host cells, together with subsequent invasion processes, can impact cell stiffness (Figure 3A) [122]. Following *Shigella* invasion, F-actin expression in the cytoskeleton increases, and atomic force microscopy (AFM) shows an increase in cell stiffness. This study also indicated that this change in cell stiffness did not vary with the number of invading bacteria. Some microorganisms possess specialized secretion systems, such as the Type III Secretion System (T3SS), which can directly inject effector factors into the host cell to activate relevant signaling pathways [123]. EseQ, a novel T3SS effector of *Edwardsiella piscicida* has been proved to bind to α-tubulin and actin cytoskeletons of host cell, causing microtubule destabilization [124].

In addition to direct bacterial invasion affecting cytoskeletal changes, virulence factors secreted by bacteria, such as *Helicobacter pylori*’s CagA, can also influence the mechanical properties of cells (Figure 3B) [125]. CagA alters the F-actin expression levels in host cells, inducing cytoskeletal reorganization. Adhesion molecules may be another aspect of cancer cell biomechanics influenced by microorganisms. Jenkins et al. demonstrated that dysbiosis can modulate the adhesion molecules on cancer cells [129]. This modulation can affect suppression of tumor endothelial adhesion molecules and activated and effector CD8^+^ T cells in the tumor. The connection between E-cadherin and cytoskeleton heavily relies on its intracellular domain being connected to catenin [130]. Helicobacter pylori injects the virulence factor CagA into the host gastric epithelial cells by activating integrins, and intracellular CagA disrupts the E-cadherin-β-catenin complex [131]. However, β-catenin is the bridge connecting the intracellular domain of E-cadherin to the actin skeleton, which may induce the arrangement of cytoskeleton [132]. In addition, MARTXVc toxin produced by *Vibrio cholerae* can also induce cytoskeletal collapse and silence host cellular responses (Figure 3C) [126].

Intimin secreted by microorganisms can bind to the host cell receptor Tir, triggering multiple downstream signaling pathways. On one hand, this interaction facilitates the recruitment of N-WASP, which subsequently activates the Arp2/3 complex to promote actin filament polymerization and the formation of pedestal-like structures beneath adherent bacteria [133]. On the other hand, intimin–Tir binding may also lead to the activation of phospholipase C (PLC), resulting in inositol 1,4,5-trisphosphate (IP3) production and the release of calcium from intracellular stores [134]. This calcium signaling cascade contributes to cytoskeletal rearrangement, including F-actin polymerization and membrane ruffling. Notably, the calcium influx can also promote the internalization of bacterial components such as LPS, which in turn may activate caspase-4 and trigger pyroptosis, a form of programmed inflammatory cell death.

A range of bacterial pathogens exhibit the ability to engage host integrins. For instance, *Yersinia pseudotuberculosis* manipulates focal adhesion proteins, such as CAS and Crk, to initiate actin remodeling via Rac1 activation [135]. Similarly, *Streptococcus agalactiae* targets integrins (e.g., α3β1 and α2β1) to induce cytoskeletal changes facilitating bacterial internalization [136]. These observations support a broader view that microbial integrin engagement can profoundly impact host cellular tension, shape, and adherence—all key elements of tumor cell biomechanics. Microbial interaction with fibronectin-integrin complexes, as exemplified by *Staphylococcus aureus*, highlights an alternative biomechanical regulatory pathway [137]. The fibronectin-binding proteins (FnBPs) of this bacterium promote integrin clustering and cytoskeletal rearrangement, with functional parallels to tumor cell responses to ECM stiffness and matrix reorganization.

Both the invasion process and subsequent intercellular spread of bacteria are accompanied by cytoskeletal remodeling and alterations in cellular biomechanical properties. For example, during *Salmonella* invasion of epithelial cells, its T3SS activates multiple signaling pathways, inducing actin cytoskeletal rearrangements and membrane ruffling that facilitate bacterial internalization (Figure 3D) [127]. Moreover, the intercellular dissemination of internalized bacteria is likewise associated with dynamic cytoskeletal remodeling. During cell-to-cell spread, bacteria can induce the formation of actin-based “comet tails” at their posterior ends, while simultaneously regulating cytoskeletal-associated proteins to promote the assembly of linear F-actin, thereby driving directional bacterial motility (Figure 3E) [128].

Dynamic visualization of bacteria spread within tumors and real-time measurements of the accompanying biomechanical changes remain technically challenging. Nevertheless, the biomechanical signals generated during this process are likely to play critical roles in bacterial transport and tumor progression and therefore warrant further investigation. Further systematic investigation of microbe-induced alterations in host cellular mechanophenotypes may open new avenues for the development of innovative therapeutic strategies in cancer treatment.

### 4.2. Remodeling TME Biomechanical Landscape

Beyond regulating the biomechanics of individual cells, microorganisms can remodel the biomechanical properties of the TME at the tissue and microenvironmental levels. Studies underscore the pivotal role of microorganisms in shaping the physical properties of the TME and how these alterations impact cancer cell behavior (Table 1).

Recent studies have demonstrated that microorganisms can influence ECM, thereby reshaping the physical landscape of tumors. This influence stems from microbial metabolite secretion, which affects the cross-linking of ECM components, such as collagen and hyaluronic acid. Transcriptome data reveal that *Fusobacterium animalis* in CRC exhibits unique associations with collagen and immune related pathways [138]. *Streptococcus gallolyticus* promotes colorectal cancer progression by upregulating collagen expression and enhancing tumor cell proliferation through ECM modulation in vitro and in vivo [139]. Such increased collagen deposition directly elevates TME stiffness, a well-documented biomechanical hallmark of tumor progression that facilitates tumor cell invasion and metastasis. MMPs degrade collagen and other structures in the TME, disrupting physical barriers of the TME and reduce ECM stiffness, thereby facilitating tumor cell dissemination [85]. Notably, *Fusobacterium nucleatum*, highly abundant in CRC and breast cancer, induces MMPs secretion and accelerates cancer progression [140,141]. Bacteria possess diverse protein secretion systems, including the type II secretion system, which mediates extracellular release of lipases, metalloproteases, and digestive enzymes [142]. However, there no studies have yet demonstrated direct secretion of these enzymes by intratumoral microorganisms or their subsequent effects on the TME.

Bacterial modulation of angiogenesis represents another key mechanism through which bacteria influence the physical microenvironment of tumors. Angiogenesis, critical for tumor progression and metastasis, ensures nutrient and oxygen delivery to tumor cells [143], and also profoundly affects TME biomechanics by regulating vascular density, per-meability, and IFP. Bacteria employ varied strategies to inhibit tumor angiogenesis [144,145,146]. Some invade nascent blood vessel endothelial cells, inducing structural damage, vascular rupture, and apoptosis, thereby directly impeding angiogenesis and altering vascular topology [147]. Others produce anti-angiogenic compounds such as angiostatin and endothelial growth inhibitory protein, which suppress endothelial cell proliferation, migration, and differentiation, ultimately hindering tumor vascularization [148,149,150], and also profoundly affects TME biomechanics by regulating vascular density, permeability, and interstitial fluid pressure (IFP). VEGF, a major regulator of pathological vascular development, is secreted by fibroblasts, inflammatory cells, and tumor cells. *Salmonella* exerts anti-angiogenic effects by downregulating VEGF expression, potentially via upregulation of gap junction protein Cx43 [151,152].

Microorganisms HA in the ECM while concurrently altering fluid stress in the TME. Kim et al. engineered an ECM-targeting *Salmonella typhimurium*, which exhibits higher hyaluronidase (HAase) activity than that derived from other strains [153]. Bacterial HAase secretion reduced IFP in the TME, enhancing chemotherapy drug penetration.

**Table 1:** Summary of current understanding of the role of microorganisms in remodeling TME biomechanical landscape.

Bacterial Species	Mechanism	Main Contributor	Consequence	Reference
*Fusobacterium animalis*	Alters ECM composition via collagen-associated signaling	FadA	Promotes ECM remodeling and immune modulation pro-tumorigenesis	[138]
*Streptococcus gallolyticus* subsp. *gallolyticus*	Induces collagen synthesis	MMP-mediated ECM degradation	Enhances ECM deposition pro-tumorigenesis	[139]
*Fusobacterium nucleatum*	Stimulates MMP expression in tumor milieu	MMP-mediated ECM degradation	Breaks ECM barriers pro-tumorigenesis	[140,141]
*Salmonella* spp.	Suppresses VEGF expression via gap junction protein Cx43	Cx43 upregulation	Inhibits angiogenesis, altering vascular topology and perfusion anti-tumorigenesis	[151,152]
Engineered *Salmonella typhimurium ATCC 29213*	Secretes hyaluronidase targeting ECM hyaluronic acid	HAase activity enhancement	Reduces IFP, enhancing drug delivery into tumors anti-tumorigenesis	[153]

## 5. Application and Prospect of Tumor Microorganisms

**Figure 4:**
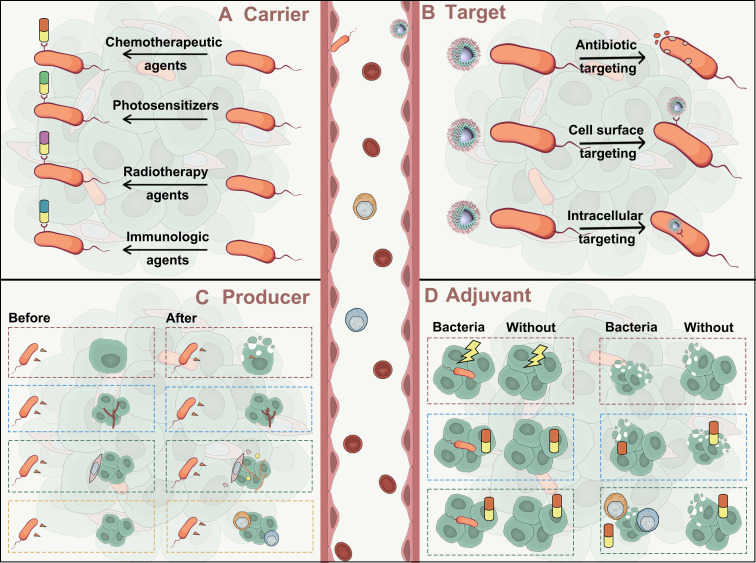
The application strategies of microorganisms in tumor therapy. (**A**) As carriers, engineered bacteria delivers chemotherapeutics, photosensitizers, radiosensitizers, and immunotherapeutic agents; (**B**) As target, they enable precise targeting Antibiotic-based, surface molecules, intracellular of bacteria in TME; (**C**) As producers, they secrete cytotoxic proteins, angiogenesis regulators, ECM modifiers, and immune modulators; (**D**) As adjuvants, they enhance radiotherapy, chemotherapy, and immunotherapy. These strategies highlight microbial precision in modulating tumor mechanics, drug delivery, and immune-microenvironment crosstalk.

The application of microorganisms in tumor therapy can be traced back to the late 19th century. In 1891, William B. Coley injected *Streptococcus* into the tumor of a patient who was unsuitable for surgery, and observed tumor regression [154]. Advances in immunology and synthetic biology have since enabled the development of microbial-vectored cancer vaccines. For instance, the Bacillus Calmette–Guérin vaccine, originally developed for tuberculosis prevention, has been clinically used in bladder cancer treatment for over 50 years [155]. Microorganisms can be applied in tumor therapy through four primary strategies: “carrier”, “target”, “producer” and “adjuvant” (Figure 4).

### 5.1. Microorganisms Act as Carrier

**Figure 5:**
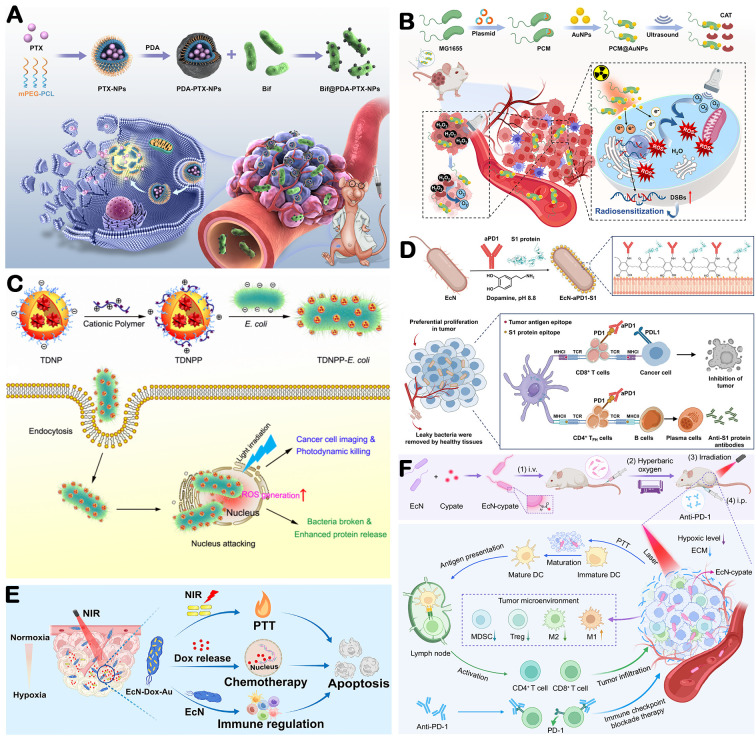
Microorganisms act as Carrier. (**A**) Schematic diagram shows the construction of the Bif@PDA-PTX-NPs biohybrid and its intelligent responsibility to reductive tumor microenvironment through self-driven targeting to hypoxic regions of tumor. Adapted with permission from Ref. [159]. Copyright 2022, DOVE Medical Press. (**B**) Preparation steps of PCM@AuNPs. Adapted with permission from Ref. [160]. Copyright 2025, Springer Nature. (**C**) Intracellular trafficking of nanoparticle-coated live *E. coli* and PS delivery. Adapted with permission from Ref. [161]. Copyright 2019, American Chemical Society. (**D**) Schematic illustration of bacteria that are dressed with a hybrid immunoactive nanosurface to elicit dual anticancer and antiviral immunity. Adapted with permission from Ref. [164]. Copyright 2022, Wiley-VCH. (**E**) Schematic illustration of the developed EcN-Dox-Au microrobots for synergistic photothermal-chemotherapy and immunomodulatory effect of breast tumor. Adapted with permission from Ref. [167]. Copyright 2023, Elsevier. (**F**) Schematic illustration of HBO-enhanced intratumoral delivery of engineered bacteria for photothermal immunotherapy. Adapted with permission from Ref. [168]. Copyright 2024, Springer Nature.

Bacteria serve as effective biological vectors for anti-tumor drugs and genes due to their innate tumor-targeting ability and capacity to thrive in hypoxic, acidic conditions [156]. Bacterial surfaces contain functional groups (e.g., thiol and amino groups), enabling conjugation with chemotherapeutics or nanoparticle-encapsulated drugs for achieving drug loading onto the bacterial surface [157]. For example, doxorubicin has been linked to amino groups on *Escherichia coli* surfaces for targeted tumor therapy [158]. *Bifidobacterium infantis* was designed to deliver paclitaxel nanoparticles to tumor tissues (Figure 5A) [159]. Beyond chemotherapy, bacteria can also deliver radiosensitizers (Figure 5B) and photosensitizers (Figure 5C) to enhance anti-tumor efficacy [160,161]. Liu et al. demonstrated that photosensitizer-loaded nanoparticles attached to bacteria generate reactive oxygen species (ROS) upon laser irradiation, inhibiting tumor growth and preventing tumor recurrence [162]. Pan et al. engineered bacteria to release radiosensitizer in response to elevated MMP-2 in the ECM [163]. This approach extends to immunotherapy (Figure 5D) [164]. Chowdhury et al. designed a nonpathogenic *E. coli* strain to selectively target and undergo autolysis within the TME, releasing a CD47-blocking nanobody that reverses immunosuppression and prolongs survival [165]. Gurbatri et al. further augmented anti-tumor efficacy by engineering bacteria to controllably express antiPD-1 and antiCTLA-4 [166]. Engineered bacteria can also be designed to carry multiple therapeutic payloads, such as photosensitizers and chemotherapeutic agents (Figure 5E,F) [167,168]. In combination with photodynamic therapy, these engineered bacteria enable the integration of chemotherapy and immunotherapy, thereby synergistically suppressing tumor progression. As versatile drug delivery vehicles, optimized bacterial designs hold significant potential for modulating the physical TME.

### 5.2. Microorganisms Act as Target

**Figure 6:**
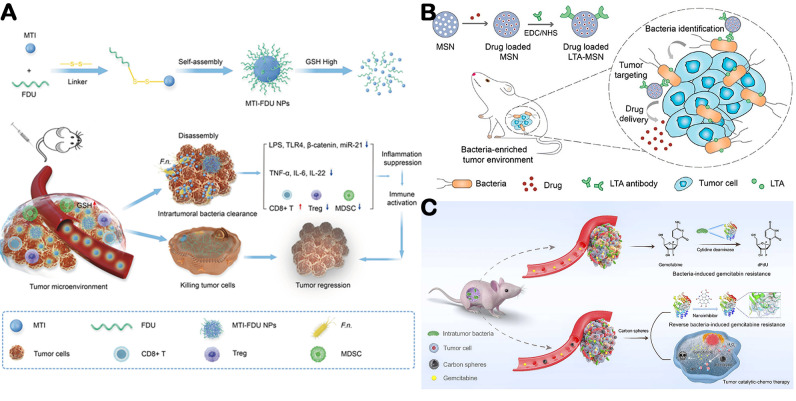
Microorganisms act as target. (**A**) Nanoparticles composed of metronidazole (MTI) as the primary component can eliminate intratumoral bacteria and promote immune infiltration. Adapted with permission from Ref. [170]. Copyright 2023, American Chemical Society. (**B**) Schematic of bacteria-guided tumor-targeting strategy for efficient tumor targeting and drug delivery. Adapted with permission from Ref. [12]. Copyright 2022, American Chemical Society. (**C**) Schematic illustration of the strategy using carbon nanospheres to overcome tumor drug-resistance induced by intratumor bacteria and synergize gemcitabine chemotherapy with nanozyme-mediated catalytic therapy for tumor treatment. Adapted with permission from Ref. [171]. Copyright 2022, Elsevier.

Intratumoral microorganisms can serve as important therapeutic targets to enhance antitumor efficacy. First, broad elimination of intratumoral microorganisms using antibiotics or nanocarrier-based delivery systems can remodel the tumor microenvironment and activate antitumor immune responses. Wang et al. developed a nano-drug delivery system that selectively targets anaerobic bacteria in the TME with antibiotics, without disrupting the intestinal microbiota [169]. Their results revealed that antibiotic treatment generated novel microbial antigens, facilitating CD8^+^ T cell recognition of tumors. The efficacy of immune checkpoint inhibitors remains limited in microsatellite-stable CRC. However, intratumoral bacteria may serve as a source of neoepitopes that could enhance immunotherapeutic approaches (Figure 6A) [170]. Beyond targeted killing of tumor microorganisms with antibiotics, the unique surface agents of bacteria can be exploited for targeted delivery (Figure 6B). Song et al. reported a delivery strategy that exploits the lipoteichoic acid as antigenic targets to modify nanoparticles with specific antibodies, thereby enabling precise targeting of intratumoral bacteria [12]. In murine models of colorectal, lung, and breast cancer, this bacteria-targeted delivery system achieved pronounced antitumor effects, highlighting the therapeutic potential of leveraging the tumor microbiome for targeted drug delivery. Third, precise modulation of critical bacterial genes or enzymes may suppress their tumor-promoting functions (Figure 6C). A nano-inhibitor of cytidine deaminase (CDD) was employed to overcome bacteria-induced gemcitabine resistance, thereby restoring tumor sensitivity to gemcitabine [171].

### 5.3. Microorganisms Act as Producers

**Figure 7:**
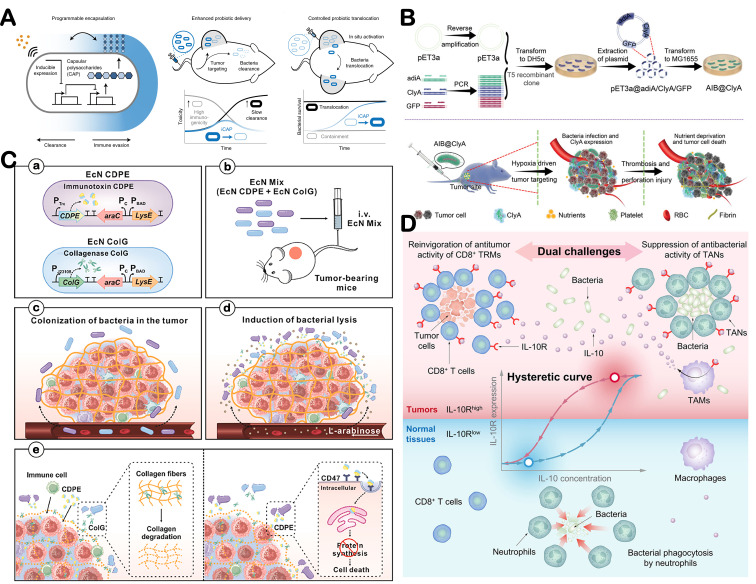
Microorganisms act as producer. (**A**) Programmable capsular polysaccharide system for control over bacterial encapsulation and in vivo delivery profiles. Adapted with permission from Ref. [175]. Copyright 2022, Springer Nature. (**B**) Schematic illustration of bacteria-triggered tumor thrombosis and the subsequent nutrient deprivation for tumor ablation in vivo. Adapted with permission from Ref. [178]. Copyright 2022, Wiley-VCH. (**C**) The mechanism of cancer therapy with engineered EcN probiotics. Adapted with permission from Ref. [179]. Copyright 2025, Elsevier. (**D**) Bacteria induce TAMs to secrete IL-10, evading TANs while activating CD8^+^ TRM cells. Adapted with permission from Ref. [181]. Copyright 2025, Elsevier.

Microorganisms possess specialized secretion systems that enable them to release effector molecules extracellularly or directly inject them into cytoplasm or plasma membrane of host cells [172]. Through these secretions, they can disrupt the TME signaling pathways and exert anti-tumor effects [173]. As producers, they secrete cytotoxic proteins, angiogenesis regulators, ECM modifiers, and immune modulators that maximize therapeutic efficacy. *E. coli* *Nissle 1917* was engineered to express the redox protein azurin, which inhibited melanoma and other cancer cell growth [174]. Azurin increases intracellular levels of p53 and Bax, promoting cytochrome c release from mitochondria into the cytosol, thereby triggering apoptosis in tumor cells. Researchers have employed a tunable microbial surface engineering strategy based on synthetic gene circuits to dynamically regulate capsular polysaccharide biosynthesis, which enhances in vivo bacterial survivability, immunogenicity, and therapeutic delivery efficiency (Figure 7A) [175]. Moreover, microorganisms can express angiogenic regulators, such as extracellular vesicles, to promote angiogenesis [176]. He et al. showed that *E. coli Nissle 1917* engineered to overexpress tumstatin suppressed melanoma growth and angiogenesis [177]. *Escherichia coli* has also been engineered to express cytolysin A protein, enhancing intratumoral thrombosis formation and thereby restricting nutrient supply to the tumor (Figure 7B) [178]. Additionally, microorganisms may modulate the extracellular matrix via enzymes such as collagenase (Figure 7C) [179]. They can also produce proteins or metabolites that modulate immune response. For example, *E. coli Nissle 1917* colonizes tumors and converts intratumoral nitrogenous compounds into L-arginine, a key metabolite for effective anti-tumor T cell responses [180]. Engineered *Salmonella enterica* can simultaneously evade antimicrobial defenses and stimulate anti-tumor immunity in the TME by driving a tumor-specific IL-10R^hi^ state in immune cells, effectively suppressing tumor growth, recurrence, and metastasis (Figure 7D) [181].

### 5.4. Microorganisms Act as Adjuvant

**Figure 8:**
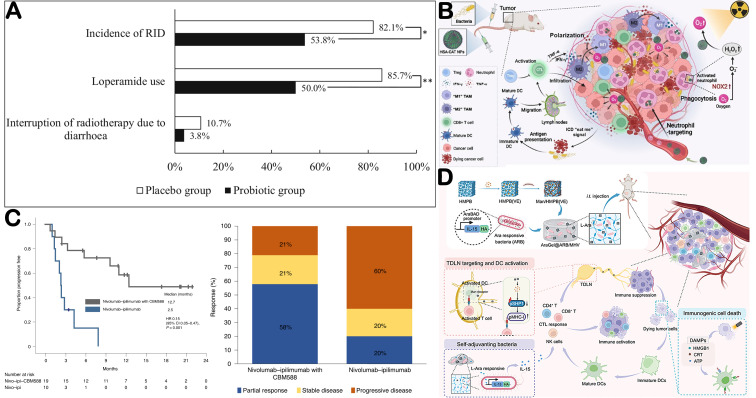
Microorganisms act as adjuvant. (**A**) Comparisons of the proportions of incidence of RID, loperamide use and interruption of radiotherapy due to diarrhoea between the probiotic group and the placebo group. Adapted with permission from Ref. [184]. Copyright 2019, Springer Nature. * *p* < 0.05, ** *p* < 0.01. (**B**) Schematic illustration of bacteria-assisted delivery and oxygen production of HSA-CAT NPs to enhance radioimmunotherapy of cancer. Adapted with permission from Ref. [185]. (**C**) Efficacy outcomes in the treatment of patients with mRCC using nivolumab–ipilimumab with or without CBM588. Adapted with permission from Ref. [186]. Copyright 2022, Springer Nature. (**D**) Schematic illustration of AraGel@ARB/MHV system for TDLN remolding, self-adjuvanting bacteria, and immunogenic cell death. Adapted with permission from Ref. [191]. Copyright 2024, WILEY-VCH.

Combining bacteria with radiotherapy can enhance tumor sensitivity to radiation, improve therapeutic efficacy, reduce side effects, and lower the required treatment dose. For example, multiple studies have demonstrated that Salmonella can augment the anti-tumor effects of radiotherapy [182,183]. The use of probiotics during radiotherapy can effectively reduce the incidence of diarrhea (Figure 8A) [184]. Ni et al. found that combining *Salmonella typhimurium* with γ-radiation suppressed tumor growth more effectively and prolonged mouse survival and boost the antitumor immunity (Figure 8B) [185]. Bacteria also hold great potential as adjuvants for chemotherapy. In a clinical trial of nivolumab plus ipilimumab for metastatic renal cell carcinoma, patients who received bifidobacterial products exhibited significantly longer progression-free survival compared with those who did not. Notably, in the control group, one patient who consumed yogurt supplemented with *Bifidobacterium animalis* showed the deepest treatment response, with an 82% reduction in tumor size (Figure 8C) [186]. *Salmonella typhimurium VNP20009* also serves as a vector for gene-directed enzyme prodrug therapy by converting inactive prodrugs into active cytotoxic compounds via cytosine deaminase, nitroreductase, or purine nucleoside phosphorylase [187]. Bacteria can also act synergistically with immunotherapy. In melanoma therapy, the symbiotic bacterium *Bifidobacterium longum* has been shown to increase CD8^+^ T cell infiltration into the TME, enhancing the anti-tumor efficacy of PD-1/PD-L1 blockade [188]. Similarly, *Akkermansia muciniphila* has been associated with improved clinical responses to PD-1 immunotherapy in patients with non-small-cell lung cancer and renal cancer patients [189,190]. Engineered bacteria can also respond to arabinose released from hydrogel degradation, thereby enhancing the efficacy of immunotherapy (Figure 8D) [191].

Despite the promising applications of microorganisms in cancer therapy, several challenges hinder their clinical implementation, including significant interpatient and intertumoral variability, safety concerns (e.g., host immune responses and infections from microbial overgrowth), and the low abundance of intratumoral microorganisms that complicates targeting and activation of intratumoral therapies. These challenges highlight critical priorities for future research. They include personalized diagnostics, innovative bacterial antitumor therapies, and targeted microbial engineering. Therefore, understanding the interactions between tumor-associated microbes and physical TME is essential for elucidating tumor progression mechanisms, identifying novel prognostic biomarkers and therapeutic targets, and optimizing diagnostic and therapeutic strategies. From the biomechanical perspective, the future research directions of tumor microorganisms can be focused on three core aspects, each with distinct advantages and limitations. First, the precise identification of microbial species that regulate tumor biomechanics. This direction has the advantage of strong targeting, which can directly lock in key functional microorganisms and lay a foundation for subsequent targeted intervention. However, it requires large-scale sample verification and high-throughput sequencing technology support. Second, the molecular mechanism of microbial regulation of tumor cell mechanical properties (stiffness, deformability, contractility). It can deeply reveal the interaction mode between microbes and tumor cells, providing clear molecular targets for therapy. The complex regulatory network involves multiple signaling pathways and demands elaborate in vitro and in vivo validation models. Third, the development of therapeutic strategies based on microbial remodeling of TME biomechanics. This direction has strong clinical transformation potential, but the disadvantage is that it needs to balance the safety and effectiveness of microbial intervention.

## 6. Conclusions

Tumor-associated microorganisms represent a pivotal frontier in cancer research. Studies have provided direct experimental evidence that intracellular bacteria can reprogram the host cell cytoskeleton. This microbial-driven remodeling enhances tumor cell resistance to shear stress in the bloodstream and facilitates distant organ colonization, revealing the mechanism of bacteria in metastatic progression. This interplay introduces a novel dimension to our understanding of cancer pathophysiology.

However, the direct mechanistic links defining how specific microbes govern tumor cell mechanics (stiffness, deformability, and contractility), and control tissue-scale physical properties (solid stress and interstitial fluid pressure) remain markedly underexplored. This gap constitutes a critical deficiency in our current knowledge. Research in this domain is nascent, with most insights inferred from indirect associations. A dedicated, systematic investigation into the causative biomechanical influence of the tumor microbiome is therefore urgently needed. Unraveling these mechanisms will not only provide fundamental insights into a previously overlooked driver of tumor progression and metastasis but also pave the way for groundbreaking therapeutic strategies. By elucidating how microbes reshape the physical tumor landscape, we can pioneer innovative interventions. These could include engineered microbial agents or anti-virulence therapies aimed at normalizing the tumor biomechanical microenvironment to suppress invasion, enhance drug delivery, and overcome treatment resistance.

In summary, while the biomechanical impact of tumor microorganisms is a compelling and likely crucial aspect of cancer biology, it remains a nascent field ripe for discovery. Prioritizing this research direction holds exceptional promise, offering a powerful new lens through which to decipher tumor biology and innovate in cancer therapy.

## Data Availability

No new data were created or analyzed in this study. Data sharing is not applicable to this article.

## Abbreviations

The following abbreviations are used in this manuscript:

CAFS	cancer-associated fibroblasts
CDD	cytidine deaminase
CEACAM1	carcinoembryonic antigen-related cell adhesion molecule 1
ECM	extracellular matrix
IFP	interstitial fluid pressure
IL	interleukin
LPS	lipopolysaccharides
MMPs	matrix metalloproteinases
ROCK	Rho-associated protein kinase
SCFAs	Short-chain fatty acids
TIGIT	T cell immunoreceptor with Ig and ITIM domains
TME	tumor microenvironment
VEGF	vascular endothelial growth factor
